# Supplementary material for: Machine Learning Accelerates Crystallization for Structure Determination
Source: Angew Chem Int Ed Engl. 2026 May 4;65(25):e1218503. doi: 10.1002/anie.1218503 (PMC13266958; doi:10.1002/anie.1218503)

checkCIF/PLATON report

Structure factors have been supplied for datablock(s) lcz089

THIS REPORT IS FOR GUIDANCE ONLY. IF USED AS PART OF A REVIEW PROCEDURE FOR PUBLICATION, IT SHOULD NOT REPLACE THE EXPERTISE OF AN EXPERIENCED CRYSTALLOGRAPHIC REFEREE.

No syntax errors found. CIF dictionary Interpreting this report

Datablock: lcz089

Bond precision:	C-C = 0.0054 A		Wavelength=1.54184
Cell:	a=13.5630(1)	b=11.3574(1)	c=22.5840(1)
	alpha=90	beta=92.557(1)	gamma=90
Temperature:	100 K		

	Calculated	Reported
Volume	3475.39(4)	3475.38(4)
Space group	P 21/n	P 1 21/n 1
Hall group	-P 2yn	-P 2yn
Moiety formula	C15 H3 Ag3 F18 N6, C14 H11 N O S	C15 H3 Ag3 F18 N6, C14 H11 N O S
Sum formula	C29 H14 Ag3 F18 N7 O S	C29 H14 Ag3 F18 N7 O S
Mr	1174.14	1174.14
Dx,g cm-3	2.244	2.244
Z	4	4
Mu (mm-1)	15.269	15.269
F000	2256.0	2256.0
F000'	2267.76	
h,k,lmax	17,14,28	16,14,28
Nref	7420	7167
Tmin,Tmax	0.064,0.217	0.175,1.000
Tmin'	0.004	

Correction method= # Reported T Limits: Tmin=0.175 Tmax=1.000
AbsCorr = MULTI-SCAN

Data completeness= 0.966 Theta(max)= 77.783

R(reflections)= 0.0400(6809) wR2(reflections)= 0.1148(7167)

S = 1.056 Npar= 533

The following ALERTS were generated. Each ALERT has the format
test-name_ALERT_alert-type_alert-level.
Click on the hyperlinks for more details of the test.

Alert level C

```
PLAT911_ALERT_3_C Missing FCF Refl Between Thmin & STh/L=    0.600          6 Report
             -11 10   2,   10   0   4,    5   8   6,    7 11   9,  -2 11 10,    7 11 10,
PLAT971_ALERT_2_C Check Calcd Resid. Dens.  1.00Ang From Ag02            1.75 eA-3
PLAT971_ALERT_2_C Check Calcd Resid. Dens.  1.03Ang From Ag01            1.74 eA-3
PLAT971_ALERT_2_C Check Calcd Resid. Dens.  0.94Ang From Ag02            1.64 eA-3
PLAT971_ALERT_2_C Check Calcd Resid. Dens.  1.08Ang From Ag03            1.61 eA-3
PLAT971_ALERT_2_C Check Calcd Resid. Dens.  1.06Ang From Ag01            1.56 eA-3
PLAT971_ALERT_2_C Check Calcd Resid. Dens.  0.89Ang From Ag03            1.52 eA-3
```

Alert level G

```
PLAT007_ALERT_5_G Number of Unrefined Donor-H Atoms .............          1 Report
             H00U
PLAT142_ALERT_4_G s.u. on b - Axis Small or Missing .............    0.00010 Ang.
PLAT143_ALERT_4_G s.u. on c - Axis Small or Missing .............    0.00010 Ang.
PLAT153_ALERT_1_G The s.u.'s on the Cell Axes   are Equal ..(Note)     0.0001 Ang.
PLAT242_ALERT_2_G Low    'MainMol' Ueq as Compared to Neighbors of       C01C Check
PLAT242_ALERT_2_G Low    'MainMol' Ueq as Compared to Neighbors of       C01E Check
PLAT242_ALERT_2_G Low    'MainMol' Ueq as Compared to Neighbors of       C01F Check
PLAT242_ALERT_2_G Low    'MainMol' Ueq as Compared to Neighbors of       C01I Check
PLAT242_ALERT_2_G Low    'MainMol' Ueq as Compared to Neighbors of       C01L Check
PLAT242_ALERT_2_G Low    'MainMol' Ueq as Compared to Neighbors of       C01N Check
PLAT720_ALERT_4_G Number of Unusual/Non-Standard Labels ..........         73 Note
             Ag01    Ag02    Ag03    S004    F005    F006    F007    F008
             F009    F00A    F00B    F00C    F00D    F00E    F00F    F00G
             F00H    F00I    F00J    F00K    F00L    F00M    N00N    N00O
             O00P    N00Q    N00R    N00S    N00T    N00U    H00U    C00V
             C00W    H00W    C00X    C00Y    H00Y    C00Z    H00Z    C010
             H010    C011    H011    C012    H012    C013    H013    C014
             C015    C016    C017    H017    C018    C019    C01A    C01B
             H01B    C01C    C01D    C01E    C01F    C01G    C01H    C01I
             C01J    H01A    H01C    H01D    C01K    H01K    C01L    C01M
             C01N
PLAT883_ALERT_1_G Absent Datum for _atom_sites_solution_primary ..     Please Do !
PLAT910_ALERT_3_G Missing FCF Reflection(s) Below Theta(Min)[Deg]=       3.88 Note
              -1   0   1,
PLAT912_ALERT_4_G Missing # of FCF Reflections Above STh/L=  0.600        230 Note
PLAT941_ALERT_3_G Average HKL Measurement Multiplicity ..........        3.1 Low
PLAT969_ALERT_5_G The 'Henn et al.' R-Factor-gap value ..........      3.422 Note
             Predicted wR2: Based on SigI**2  3.35 or SHELX Weight 10.87
PLAT978_ALERT_2_G Number C-C Bonds with Positive Residual Density.          0 Info
```

```
   0 ALERT level A = Most likely a serious problem - resolve or explain
   0 ALERT level B = A potentially serious problem, consider carefully
   7 ALERT level C = Check. Ensure it is not caused by an omission or oversight
  17 ALERT level G = General information/check it is not something unexpected

   2 ALERT type 1 CIF construction/syntax error, inconsistent or missing data
  13 ALERT type 2 Indicator that the structure model may be wrong or deficient
   3 ALERT type 3 Indicator that the structure quality may be low
   4 ALERT type 4 Improvement, methodology, query or suggestion
   2 ALERT type 5 Informative message, check
```

Validation response form

Please find below a validation response form (VRF) that can be filled in and pasted into your CIF.

```
# start Validation Reply Form
_vrf_PLAT911_lcz089
;
PROBLEM: Missing FCF Refl Between Thmin & STh/L=    0.600          6 Report
RESPONSE: ...
;
_vrf_PLAT971_lcz089
;
PROBLEM: Check Calcd Resid. Dens.  1.00Ang From Ag02            1.75 eA-3
RESPONSE: ...
;
# end Validation Reply Form
```

It is advisable to attempt to resolve as many as possible of the alerts in all categories. Often the minor alerts point to easily fixed oversights, errors and omissions in your CIF or refinement strategy, so attention to these fine details can be worthwhile. It is up to the individual to critically assess their own results and, if necessary, seek expert advice.

PLATON version of 17/09/2025; check.def file version of 17/09/2025

duplicate check

No duplication found

Datablock lcz089 - ellipsoid plot

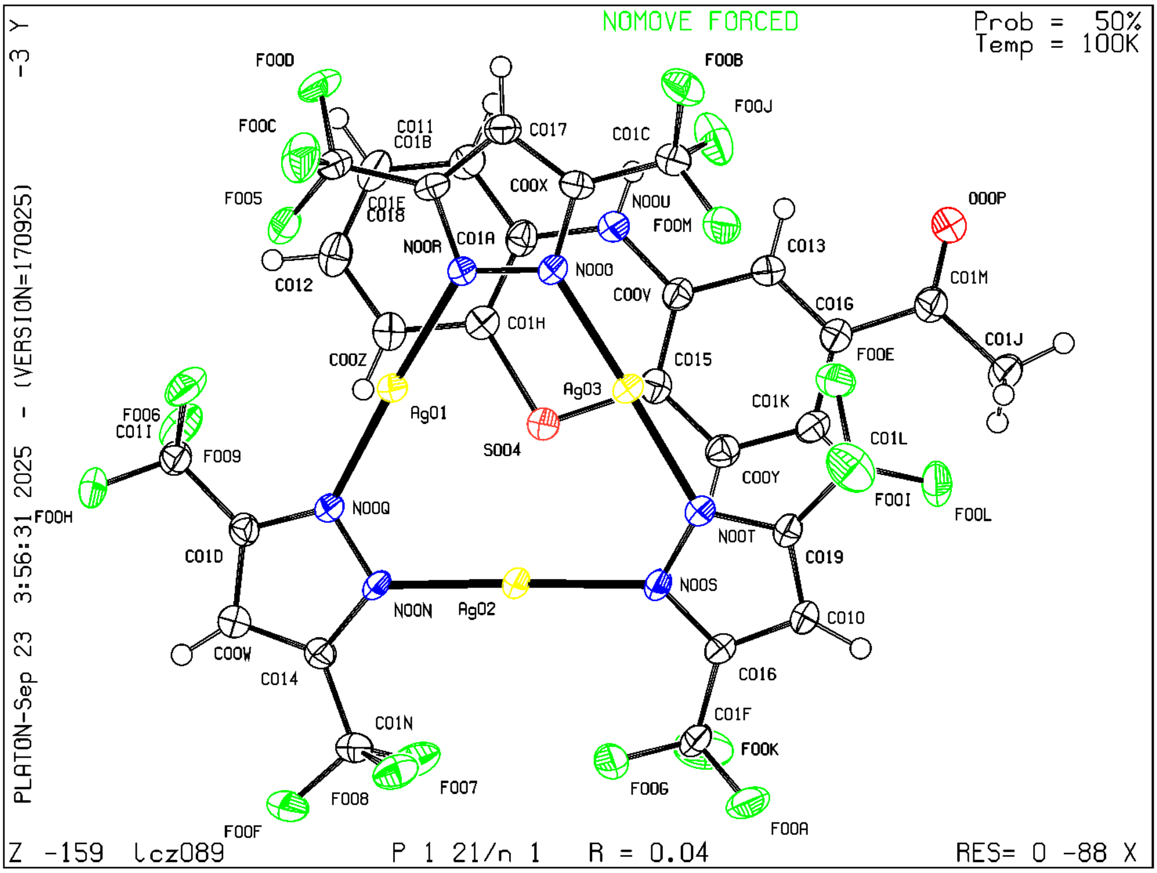

Supplement: Supplementary file 2 — Supporting File 2: anie72462‐sup‐0002‐DataFile.zip. [file ANIE-65-e1218503-s002.zip › supplementary_materials_crystallographic_data submit.zip!/cif_and_checkcif(1-114)/Ag3Pz3_89/Ag3Pz3_89_checkcif.pdf]
